# The fungal airway microbiome in cystic fibrosis and non-cystic fibrosis bronchiectasis

**DOI:** 10.1016/j.jcf.2020.05.013

**Published:** 2021-03

**Authors:** Leah Cuthbertson, Imogen Felton, Phillip James, Michael J. Cox, Diana Bilton, Silke Schelenz, Michael R. Loebinger, William O.C. Cookson, Nicholas J. Simmonds, Miriam F. Moffatt

**Affiliations:** a Royal Brompton and Harefield NHS Foundation Trust, Sydney Street, London SW3 6NP, UK; b National Heart and Lung Institute, Imperial College, London SW3 6LY, UK

**Keywords:** Mycobiome, Fungal airway disease, Filamentous fungi, Chronic airway disease

## Abstract

**Background:**

The prevalence of fungal disease in cystic fibrosis (CF) and non-CF bronchiectasis is increasing and the clinical spectrum is widening. Poor sensitivity and a lack of standard diagnostic criteria renders interpretation of culture results challenging. In order to develop effective management strategies, a more accurate and comprehensive understanding of the airways fungal microbiome is required. The study aimed to use DNA sequences from sputum to assess the load and diversity of fungi in adults with CF and non-CF bronchiectasis.

**Methods:**

Next generation sequencing of the ITS2 region was used to examine fungal community composition (n = 176) by disease and underlying clinical subgroups including allergic bronchopulmonary aspergillosis, chronic necrotizing pulmonary aspergillosis, non-tuberculous mycobacteria, and fungal bronchitis. Patients with no known active fungal disease were included as disease controls.

**Results:**

ITS2 sequencing greatly increased the detection of fungi from sputum. In patients with CF fungal diversity was lower, while burden was higher than those with non-CF bronchiectasis. The most common operational taxonomic unit (OTU) in patients with CF was *Candida parapsilosis* (20.4%), whereas in non-CF bronchiectasis sputum *Candida albicans* (21.8%) was most common. CF patients with overt fungal bronchitis were dominated by *Aspergillus* spp.*, Exophiala* spp., *Candida parapsilosis* or *Scedosporium* spp.

**Conclusion:**

This study provides a framework to more accurately characterize the extended spectrum of fungal airways diseases in adult suppurative lung diseases.

## Highlights

• The prevalence of fungal disease is increasing in CF and non-CF bronchiectasis.
• Effective management of fungal disease requires an understanding of the mycobiome.
• Culture methods alone are inadequate for the accurate diagnosis of fungal disease.
• Our study provides a framework to characterize fungal airway disease using NGS.
• NGS can improve detection and clinical management of fungal infections.

## 1 Introduction

Cystic fibrosis (CF) and non-CF bronchiectasis are chronic suppurative lung diseases characterised by permanent bronchial dilatation, variable mucociliary clearance and recurrent infections [1,2]. Recent culture-independent studies have illustrated a far more complex bacterial airway biodiversity than revealed by conventional microbiology [3]. By contrast the extent of fungal infection of the airways is much less clear, and the prevalence and clinical significance of fungal communities in sputum is comparatively poorly understood [4,5]. In addition, emergent non-*Aspergillus* filamentous fungal species, such as *Scedosporium* spp. and *Exophiala dermatitidis* have been increasingly observed in CF sputa [6,7]*.*

Several studies have recognised that the presence of fungi in airway secretions are associated with accelerated clinical decline [8], [9], [10]. In adults with CF, *Candida, Aspergillus* and non-*Aspergillus* filamentous fungi have all been associated with worse disease and significantly impaired lung function [10], [11], [12], [13]. The rising prevalence and variety of fungi in the sputum of CF patients and their association with severe disease suggest that fungi may directly contribute to airway inflammation, lung function decline and exacerbation rates [8,12].

Although patients are currently categorised according to fungal culture results, agreed standards for diagnosis are lacking [14]. This is in part due to the difficulties of fungal culture. The requirements needed to capture the range of fungal taxa are demanding as the growth rates of pathogenically relevant taxa may be slow [15] and a wide range of morphologies attributable to a single species make definitive identification difficult [16]. Therefore, a culture-independent investigation of fungal species in both CF and non-CF bronchiectasis is desirable.

Here we describe the fungal community composition in patients with CF and non-CF bronchiectasis, assessed by next generation sequencing of the polymorphic ITS2 region. We have included the recognised clinical diagnostic categories of allergic bronchopulmonary aspergillosis (ABPA), chronic necrotizing pulmonary aspergillosis (CNPA) and fungal bronchitis (FB). We have also included patients with non-tuberculosis mycobacterial infection (NTM), as there are suggested links between NTM disease and infection with *Aspergillus* and other fungal species [17,18]. A control group of patients not undergoing treatment for fungal disease was classified as the “no active fungal disease” (NAFD).

In our analyses, we provide data on the prevalence and abundance of the most common taxa within these sub-groups and test whether the sub-groups have distinct community compositions.

## 2 Methods

### 2.1 Full details of methods can be found in the supplementary methods

This was a prospective, cross-sectional study of adult patients (>16years) involving 42 cases of non-CF bronchiectasis and 134 patients with CF (Table 1). Subjects with CF were diagnosed according to standard criteria, a sweat chloride level ≥ 60 mmol/L and/or two CF-causing mutations. Patients with non-CF bronchiectasis were screened according to the British Thoracic Society bronchiectasis guidelines with subjects defined as having symptoms of chronic cough, sputum production and malaise related to airway wall thickening and dilatation. Participants with evidence of CF, primary ciliary dyskinesia or underlying immunodeficiency were excluded from the non-CF bronchiectasis group.

**Table 1:** Patient demographics. data for all samples collected in the study.

	Non-CF bronchiectasis (n = 42)	CF (n = 134)	P
Sequencing positive (over 1000 reads) (%)	32 (76.2)	114 (85.1)	0.271
**Clinical Parameters:**			
Age/yrs (mean (SD))	58.69 (11.31)	31.57 (11.02)	<0.001
Male (%)	14 (33.3)	69 (51.5)	0.06
BMI (mean (SD))	25.98 (10.39)	22.63 (8.86)	0.054
FEV1% predicted (mean (SD))	63.14 (26.10)	50.59 (19.55)	0.001
FVC% predicted (mean (SD))	87.19 (31.64)	75.75 (21.87)	0.01
Total IgE (mean (SD))	664.88 (1536.44)	209.97 (324.99)	0.008
*Aspergillus* specific IgG (mean (SD))	62.10 (48.03)	74.29 (49.22)	0.232
*Aspergillus* specific RAST (mean (SD))	8.03 (12.54)	4.20 (6.78)	0.03
C reactive Protein (mean (SD))	22.34 (53.26)	22.30 (31.14)	0.995
**Genotype:**			
Phe508del Homozygous (%)	NA	59 (44.7)	
Phe508del Heterozygous (%)	NA	61 (46.2)	
Other (%)	NA	12 (9.1)	
CFRD (%)	NA	39 (29.1)	<0.001
PI (%)	NA	115 (85.8)	<0.001
**Long term medications:**			
Anti-pseudomonal (PsA colonised) (%)	11 (26.2)	109 (81.3)	<0.001
Azithromycin (%)	13 (31.0)	88 (65.7)	<0.001
Anti-fungal (%)	18 (42.9)	46 (34.3)	0.413
NTM treatment (mean (SD))	0.02 (0.15)	0.07 (0.26)	0.238
Inhaled corticosteroids (%)	31 (73.8)	106 (79.1)	0.611
**Clinical sub-groups:**			
Allergic bronchopulmonary aspergillosis (ABPA) (%)	15 (35.7)	24 (17.9)	
Chronic necrotizing pulmonary aspergillosis (CNPA) (%)	7 (16.7)	0 (0.0)	
Fungal bronchitis (FB) (%)	0 (0.0)	29 (21.6)	
No active fungal disease (NAFD) (%)	12 (28.6)	66 (49.3)	
Non-tuberculous mycobacteria (NTM) (%)	8 (19.0)	15 (11.2)	

The study was approved by the Royal Brompton and Harefield Hospital Biomedical Research Unit Ethics Committee (Advanced Lung Disease Biobank Study Number: 10/H0504/9). Study subjects were partitioned into *a priori* defined clinical subgroups according to the study criteria listed in the on-line supplement (Table S1). The recognized term ‘*Aspergillus* Bronchitis’ was expanded to ‘Fungal Bronchitis (FB)’ to include patients who have an identical syndrome to *Aspergillus* Bronchitis but in whom filamentous fungi other than *Aspergillus* spp. are isolated from their sputum.

### 2.2 Sample collection

Paired spontaneous sputum samples were collected into sterile containers. The first sample was allocated for routine clinical microbiological culture (see Supplementary Methods). The second sample was split into 300μl aliquots under sterile conditions and stored at -80°C within 60 minutes of collection and allocated for ITS2 gene sequencing.

### 2.3 DNA extraction

DNA extraction was performed on 300μl aliquots of sputum using a DNA fast spin kit for soil (MPBio, California, USA) according to the manufacturer's instructions. A blank extraction control, comprising of sterile water, was performed for each lot number of DNA extraction kits used. DNA extraction kit controls were blinded and processed in a random order alongside patient samples.

### 2.4 Fungal 18S rRNA gene qPCR

Fungal burden was estimated using a modified Taqman based qPCR assay described in Lui *et al.* 2012 [19].

### 2.5 ITS2 fungal community sequencing

A schematic of the methodology used is shown in Figure S1. In brief, the ITS2 region was amplified using dual indexed pan-fungal primers (Table S3) alongside blinded DNA extraction kit controls; negative PCR controls and a synthetic mock fungal community of known composition (Table S4). Amplicons were purified then quantified prior to equimolar pooling. Gel extracted libraries were quality checked using a Bioanalyzer and quantified using qPCR. Bi-directional 300 bp reads were generated for each sample using an Illumina MiSeq bench top sequencer with custom sequencing primers (Table S5). Sequences were submitted to the European Nucleotide Database, project number PRJEB33434.

### 2.6 Sequence processing

In brief, Trim Galore, QIIME 1.9.0 and BWA were used alongside the R package Phyloseq [20] to produce a high quality BIOM formatted operational taxonomic unit (OTU) table. Taxonomic information was assigned to each OTU using the RDP naive Bayesian classifier trained using the UNITE database.

Comparison of culture results with the presence or absence of particular OTUs was carried out on the full dataset. In order to minimise bias while using ecological measures, 30 samples with less than 1000 reads were then removed from further analysis (Table S7). The remaining 146 (83%) of samples consisted of 32 cases of non-CF bronchiectasis and 114 cases of CF. Rarified data, samples normalised to the minimum number of reads present in samples with more than 1000 reads (n = 1008 reads) by randomised resampling, was used to estimate community diversity characteristics and to carry out indicator species analyses. DESeq2 analyses were performed on unrarefied data, for consistency samples with <1000 reads were removed from this analysis.

### 2.7 Statistical analysis

All downstream analysis was carried out in R version 3.6.1. Detailed information regarding statistical packages and approaches, including decontamination, can be found in the Supplementary Methods. *P*-values were corrected for multiple comparisons using the Benjamini-Hochberg approach. Wilcoxon rank sum tests were used to test for significant differences between means in non-normal datasets. Where multiple groups were analyzed with non-normal response variables, Kruskal-Wallis test was used in combination with Dunns’ post hoc test to determine significant differences between groups. Spearmans correlations were used to examine relationships with continuous variables. Adonis permutational ANOVA, from the vegan package version 2.5-6 [21], was used to examine changes in fungal community similarity. Differential abundance analysis using DESeq2 (version 3.6) [22] and indicator species analysis using the package indicspecies (version 1.7.8) [23] were used to identify OTUs significantly associated with disease groups (please see further details in Supplementary Methods).

### 2.8 Role of funding source

The project was supported by the Asmarley Trust, the Wellcome Trust and the NIHR Respiratory Disease Biomedical Research Unit at the Royal Brompton and Harefield NHS Foundation Trust, Imperial College London. IF was supported by an NIHR PhD studentship.

3. Results

### 3.1 Study subject demographics and clinical characteristics

A total of 176 patients were recruited (CF n = 134; non-CF bronchiectasis n = 42). The median age in CF patients was significantly lower than non-CF bronchiectasis patients (Non-CF bronchiectasis; Mean = 58.69, SE = 1.75, CF; Mean = 31.57, SE = 0.95, *P* < 0.001) (Table 1). Percentage predicted spirometric values were also lower in CF (FEV1 % predicted; Non-CF bronchiectasis; Mean = 63.14, SE = 4.13, CF; Mean = 49.50, SE = 1.69, *P* = 0.001; FVC % predicted; Non-CF bronchiectasis; Mean = 87.19, SE = 5.0, CF; Mean = 75.75, SE = 1.89, *P* = 0.01).

A higher proportion of patients with CF were on current antibiotic treatment regimes. These included long-term nebulised anti-*Pseudomonal* antibiotics for established *P. aeruginosa* chronic colonisation (CF = 81.3%, non-CF bronchiectasis = 26.2%, *P* < 0.001) and prophylactic Azithromycin (CF = 65.7%, non-CF bronchiectasis = 31.0%, *P* < 0.001). There was no significant difference in administration of inhaled corticosteroids. Both disease groups had a similar prevalence of anti-fungal treatments that included the triazole class of drugs (itraconazole, voriconazole and posaconazole) as well as intermittent intravenous caspofungin (Table 1).

The most frequent CFTR mutation among patients with CF was Phe508del (heterozygous; n = 61, homozygous; n = 59, other CFTR mutation combinations; n = 12), 29.1% had co-existent CF-related diabetes mellitus (CFRD) and 85.8% had pancreatic insufficiency.

### 3.2 Culture comparisons with ITS2 sequencing

Culture data was unavailable for 4 of the 176 patients recruited. Of the remaining 172 individuals, 18 were positive for NTMs, 137 had positive bacterial cultures and, 65 (37.8%) were fungal culture positive.

ITS2 next generation sequencing (NGS) was used to detect fungal taxa from 176 samples. Sequencing data was obtained from all samples. After removal of likely contaminants (Supplementary Information) the number of sequences per sample ranged from 32 to 991,083. After removal of samples with less than 1000 reads (n = 30), sequencing identified 447 species belonging to 281 genera. This was in contrast to the culture results from which only four fungal species were identified. Of the 30 samples removed due to lack of reads, 4 samples were culture positive. All these samples were from CF patients.

**Fig. 1:**
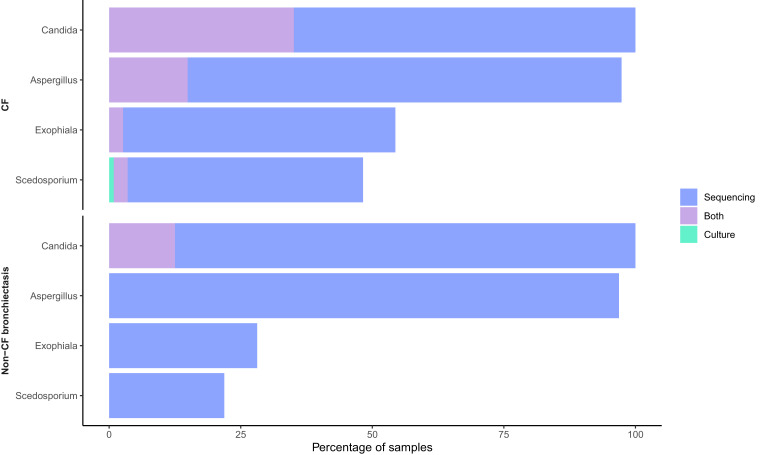
Comparison of fungal detection by culture or sequencing. The figure shows the percentage of 146 samples positive for *Scedosporium* complex, *Exophiala dermatitidis, Aspergillus* spp. or *Candida* spp. as determined by culture alone, culture and sequencing, or sequencing alone. Any sequence reads from each genus in the sample were considered positive.

In the samples with more that 1000 reads trends in detection by culture were mirrored incompletely by patterns of detection by sequencing. Samples with positive culture results were always sequence positive for the cultured taxa with the exception of a single *Scedosporium* culture positive sample (Fig. 1). *Aspergillus* spp., *Exophiala* spp. and *Scedosporium* spp. were not detected by culture in any of the non-CF bronchiectasis patients, but these organisms were identified by sequencing in 96.9%, 28.1% and 21.9% of samples respectively. These species were also cultured in the CF patients, but there was a notably higher detection rate from the sequencing approach (Fig. 1). Only 7 individuals who were culture positive for *Candida* spp. were found to be sequence positive with less than 20% of the total reads assigned to the Candida genus. This suggests that in the majority of cases culture will only identify an organism if it is dominant in the community (Figure S2).

### 3.3 Fungal taxa within disease subgroupings

**Fig. 2:**
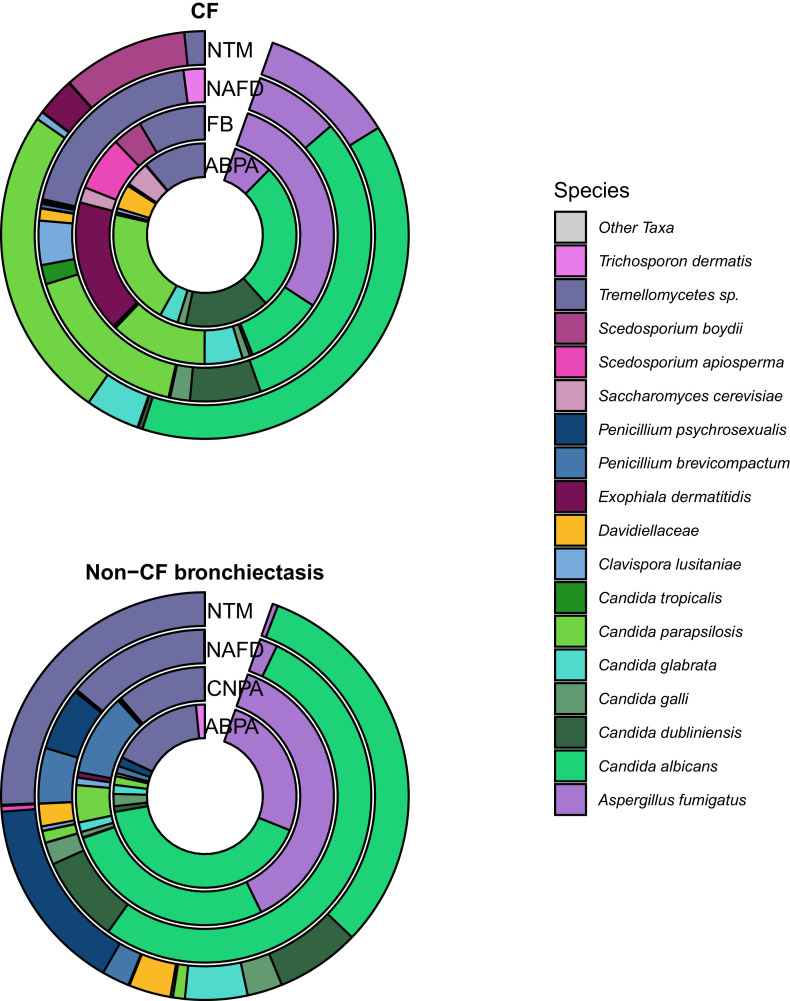
The top 20 most abundant OTUs in disease subgroups coloured by species, split by disease diagnosis. Each donut includes all samples by disease group, CF and Non-CF bronchiectasis diagnoses. Agglomerated species by clinical subgroup are indicated by rings. Within each donut, colours indicate agglomerated species identified by ITS2 sequencing. Four individual *C. albicans* OTUs are included in the top 20 OTUs within this dataset.

**Fig. 3:**
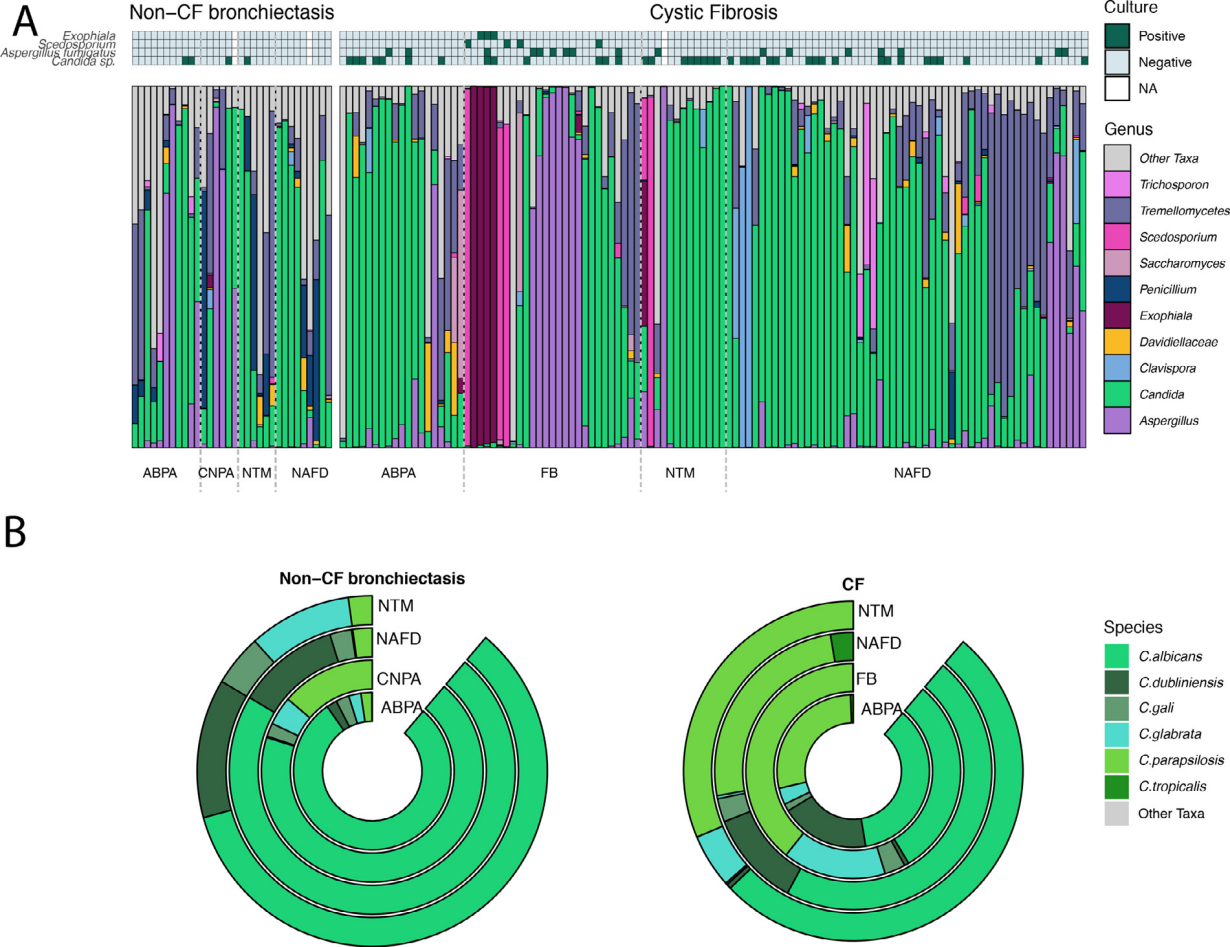
A) Fungal community composition of individual patients, Samples are grouped by disease and then subgrouped by fungal disease class. Positive fungal culture results are shown as solid boxes above the stacked bar plot of the top 20 OTUs coloured by genus. All remaining OTUs are shown in grey. B) Relative abundance of *Candida* species by disease subgroup, split by disease diagnosis. Donut plot of Candida species by disease subgroup coloured by species.

OTUs belonging to the *Candida* genus were the most prevalent and abundant across both diseases (Fig. 2). However, *Candida parapsilosis* was strikingly more prevalent and abundant in the CF disease group (W = 1189.5, *P* < 0.001) (Fig. 3). In subgroup analyses, *Candida* OTUs were significantly less abundant in those with FB compared to NAFD (*P* < 0.001) and NTM (*P* < 0.001) (Fig. 2).

Differential abundance analysis and indicator species analysis were performed on the clinical subgroups within patients with CF or non-CF bronchiectasis.

Within CF patients, indicator species analysis identified OTUs belonging to *Scedosporium apiosperma* as well as *Aspergillus fumigatus* that were significantly associated with FB (Figure S3). Differential abundance analyses showed these taxa also to be proportionally increased when compared to NAFD controls (Figure S5).

In the CF patients diagnosed as ABPA, *Aspergillus fumigatus* was less abundant and prevalent than *Candida* spp. (Fig. 3A, Figures S3) and indicator species analysis did not significantly associate *Aspergillus fumigatus* OTUs with ABPA. Furthermore, differential abundance analysis showed no significant association between the ABPA group and Aspergillus OTUs.

In patients with non-CF bronchiectasis diagnosed as ABPA, *Aspergillus fumigatus* was however the most abundant OTU. Differential abundance analysis using DESeq2 found that despite the high abundance of *Aspergillus fumigatus* OTUs in patients within the ABPA group compared with controls, when using data shrinkage the fold change observed was low and therefore more data would be required to explore this relationship (Figure S5).

In both the CF and non-CF bronchiectasis cohort, the NTM subgroup exhibited a significant increase in proportional abundance of OTUs belonging to the *Candida* genus in comparison with the NAFD controls (Figures S5).

*Aspergillus fumigatus* was the most abundant and prevalent OTU within the non-CF bronchiectasis CNPA samples and was significantly associated with this group through indicator species and differential abundance analysis (Aspergillus_fumigatus_2021; log2 fold change = 3.491, *P*-adjusted < 0.001, Aspergillus_fumigatus_3569; log2 fold change = 3.446, *P*-ajusted < 0.001, Aspergillus_fumigatus_5341; log2 fold change = 3.609, *P*-ajusted < 0.001)(Figure S4 and S5).

Differences in community composition were predominantly driven by the most abundant OTU within a sample (Fig. 3A). Shared OTUs were predominantly highlighted as Indicator taxa (Figures S3 and S4) or were found in significantly greater proportional abundance when compared with the disease matched NAFD controls (Figure S5).

### 3.4 Fungal community characteristics

**Fig. 4:**
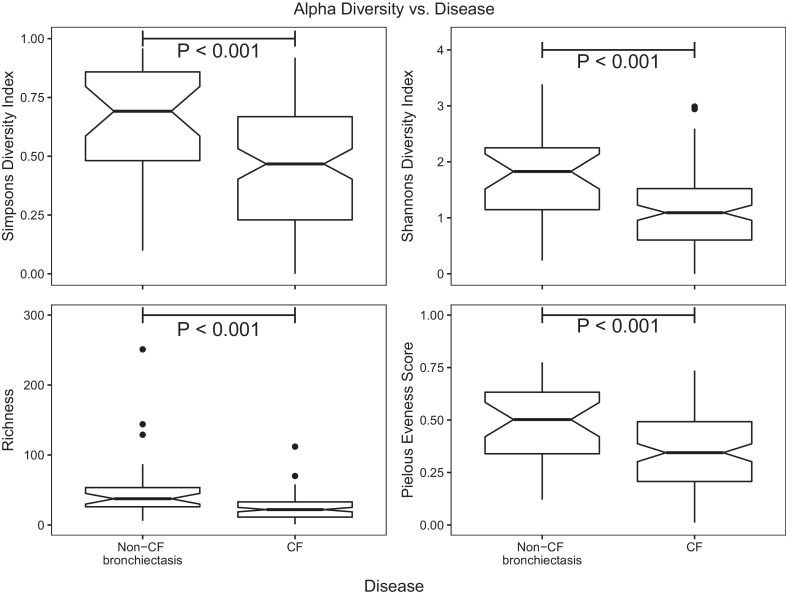
Alpha diversity of fungal communities in CF and Non-CF bronchiectasis. Significantly greater diversity was observed in samples from non-CF bronchiectasis samples compared with those from CF. The boxes encompass the data interquartile range. Whiskers denote the 75^th^ and 25^th^ percentiles respectively. Notches indicate 95% confidence interval of the median.

All fungal alpha diversity metrics were significantly reduced in the CF patient samples compared to the non-CF bronchiectasis samples (Simpsons; W = 2637, *P* < 0.001, Shannon's; = 2711, *P* < 0.001, Richness; W = 2829.5, *P* < 0.001, Evenness; W = 2475, *P* < 0.001) (Fig. 4). When comparing diversity measures amongst disease groups, patients with fungal bronchitis diagnosis were found to have a less diverse fungal community than patients with other diagnoses (Figure S6).

To highlight groups that were dominated by single OTUs, the proportion of sequences per sample assigned to the most abundant OTU in that sample was calculated (Figure S7). A significantly higher number of sequences per sample were assigned to the most dominant OTU (in that sample) in the CF patients compared with non-CF bronchiectasis (*P* < 0.001), indicating that in general the sputum of CF patients is more likely to be dominated by a particular organism.

Spearman's correlations were used to inspect trends in fungal diversity associated with changes in serological markers of fungal disease. Only age was found to have a significant positive correlation with diversity, (Richness; Spearman correlation = 0.356, *P*.adjust < 0.001, Shannon; Spearman correlation = 0.276, *P*.adjust = 0.009, Simpson; Spearman correlation = 0.239, *P*.adjust = 0.047, Pielous; Spearman correlation = 0.196, *P*.adjust = 0.248). However, age was confounded by underlying disease and a generalized linear model showed no significant relationship between alpha diversity measures and age when disease was also included as an interaction term.

There was no significant difference in 18S rRNA gene copies per g of sputum between the CF and non-CF samples (Kruskal Wallis test *P* > 0.08). However, within the CF cohort there were significantly higher copies of the fungal 18S rRNA gene per g of sputum in the FB group (*P* = 0.003) and NTM group (*P* = 0.006) compared with the no active fungal disease controls (Figure S8).

## 4 Discussion

Reports from the CF registries in the United States and the United Kingdom show a rising prevalence of airway fungal isolates in CF patients [24]. These rates are based only upon conventional cultures, although the specific culture conditions and the length of time required to detect fungal taxa are well known to hinder early detection or intervention [6]. We have shown that next generation sequencing of the ITS2 ribosomal RNA region greatly increases the detection of fungal taxa in the sputum of patients with CF and non-CF bronchiectasis. Our results suggest that the registry figures underestimate the true fungal burden in CF lungs. Furthermore, we found that sequences were also able to identify pathogenic fungi that might not be discovered through standard diagnostic surveillance.

Clinical and mechanistic studies [4,25] show a direct contribution of fungi to airway inflammation [26,27] and lung function decline [4,8,9]. Our demonstration of a substantial fungal microbiome in both CF and non-CF bronchiectasis sputum therefore identifies an important pathogenic factor which deserves further investigation.

Previous work has shown Aspergillus bronchitis to be a distinct category of fungal airways disease in CF and non-CF bronchiectasis [28]. Here we observed that emerging filamentous pathogens such as *Exophiala dermatitidis* and *Scedosporium* complex can also dominate the fungal communities supporting the concept of ‘Fungal bronchitis’. Patients with fungal bronchitis had the highest fungal biomass (Figure S8) as well as the most dominated communities (Fig. 3A and Figure S7). This clinical subgroup is often characterised by severe symptoms which prove difficult to treat. Sequencing is therefore a potentially important approach for confirming diagnosis, allowing earlier treatment and careful monitoring of progression. Longitudinal studies will be an important step in understanding the potential of improved diagnosis.

We found a marked discordance between culture and sequence in patients with chronic necrotizing pulmonary aspergillosis (CNPA). Although our molecular data confirm the presence of significant *Aspergillus* spp. burdens in these patients, it also appears to be more adept at detecting the presence of fungi. Patients with CNPA often respond to systemic antifungal therapy (although treatment may be suppressive rather than curative [29]), so that early diagnoses by sequence analysis may direct treatment and improve prognosis.

ABPA, defined as a hypersensitivity reaction to *Aspergillus*, is a frequently encountered fungal airway complication in non-CF bronchiectasis and CF [30]. Although *Aspergillus* OTUs were identified within our patients diagnosed with ABPA, *Aspergillus* spp. were not the most prevalent or abundant fungal taxa in CF patients with this diagnosis. The wide array of other fungal taxa that we also detected included a high percentage of *Candida* spp. that have been implicated as major sensitizers within the respiratory system [31]. This study provides further evidence to support the concept that other sensitising fungi may play significant roles in allergy-related complications of non-CF bronchiectasis.

The presence of *Aspergillus* spp. is considered a possible risk factor for NTM infection in CF [17] and non-CF bronchiectasis [18]. We found a single Aspergillus OTU within the top 20 OTUs in the CF-NTM group but *Candida* spp. were in the highest prevalence and abundance within these patients. Long term antibiotic treatment may be a contributing factor in promoting the growth of *Candida* spp., but further studies may be desirable to elucidate whether fungal growth is independently contributing to the lung damage of patients with NTM infections.

Our results show high prevalence and dominance of *Candida* spp. across most samples and disease groups (Fig. 3B) that is consistent with other molecular fungal profiling studies [32]. It is striking that *Candida parapsilosis* was (unexpectedly) the most abundant OTU in CF sputum (20.4%) while *Candida albicans* was most common in the group with non-CF bronchiectasis. CF airways may provide a milieu that encourages *Candida parapsilosis*, but the potential transmission of an opportunistic novel pathogen between patients warrants investigation.

*Candida* spp. are commonly cultured from patients with non-CF bronchiectasis and CF [33] and it has been suggested that *Candida* spp. may not merit therapeutic action because of “benign colonisation” or oral sample contamination [9]. To test the validity of the assumption is beyond the scope of this study. Nevertheless, *Candida* spp. are pathogenic in many circumstances. Chronic *Candida* bronchitis is associated with significant morbidity and responds well to treatment, and the presence of *Candida* spp. is associated with reduced lung function in patients with CF [9].

Our study has limitations that must be taken into consideration when interpreting our findings. The investigation is cross sectional and provides a snapshot of patients at one moment during disease progression and treatment. Microbial communities are known to be dynamic systems that change over time and longitudinal data would provide a greater insight into how the fungal microbiome alters with changes in treatment and disease state.

The subgroups into which we have classified our patients are not universally accepted as standard, in part because uniform definitions have not been agreed. In particular, our suggestion that “fungal bronchitis” be expanded beyond *Aspergillus* bronchitis to include dominant growth of other filamentous fungi is not universally accepted.

Our controls were classed as “no active fungal disease” as they were not undergoing treatment for any fungal infection. A number of these patients (n = 24) produced positive fungal cultures when first tested according to the study protocols, but were kept in the investigation to adhere to the *a priori* definitions of each group.

ITS2 amplification and sequencing yielded low numbers of reads (<1000) in 30 samples [34], so detailed comparisons of fungal taxa between patient groups was possible on 146 samples. There were however no differences in clinical characteristics between the low sequence samples and the 83% who were included in the main analyses.

## 5 Conclusions

The ecological characteristics that shape and define fungal communities within CF and non-CF bronchiectasis airways are complex. However, we have illustrated fungal airway community characteristics corresponding to a continuum from saprophytic colonisation, infective bronchitis to semi-invasive disease, and that allergic airways hypersensitivity to fungi is not a simple correlate of fungal burden. The fungal communities range from diverse and dissimilar microbiota to narrow and highly dominated communities associated with a high fungal biomass.

Despite the established significance of *Aspergillus*, our results indicate that other dominant members of the fungal airway microbiome should be considered in clinical management. In particular the role of *Candida* should be further investigated.

Finally, culture methods alone do not seem adequate for the clinical management of fungal disease. Our study exemplifies sequence-based methodologies that can be used to accurately identify fungal pathogens in adult patients with CF and non-CF bronchiectasis.

## CRediT authorship contribution statement

**Leah Cuthbertson:** Formal analysis, Investigation, Methodology, Resources, Visualization, Writing - original draft, Writing - review & editing. **Imogen Felton:** Conceptualization, Data curation, Investigation, Resources, Funding acquisition, Methodology, Writing - original draft. **Phillip James:** Formal analysis, Investigation, Methodology, Writing - original draft, Visualization. **Michael J. Cox:** Investigation, Methodology. **Diana Bilton:** Resources. **Silke Schelenz:** Investigation, Methodology. **Michael R. Loebinger:** Conceptualization, Resources, Supervision. **William O.C. Cookson:** Conceptualization, Resources, Funding acquisition, Supervision, Writing - original draft, Writing - review & editing. **Nicholas J. Simmonds:** Conceptualization, Resources, Funding acquisition, Supervision, Writing - review & editing. **Miriam F. Moffatt:** Conceptualization, Resources, Funding acquisition, Supervision, Writing - review & editing.

## Declaration of Competing Interests

The authors have no conflicts of interests to disclose.

## Appendix Supplementary materials

Image, application 1
