# Supplementary material for: The fungal airway microbiome in cystic fibrosis and non-cystic fibrosis bronchiectasis
Source: J Cyst Fibros. 2021 Mar;20(2):295–302. doi: 10.1016/j.jcf.2020.05.013 (PMC8048771; doi:10.1016/j.jcf.2020.05.013)
Supplement: Supplementary file 1 [file mmc1.docx]

**Supplementary Material**

The Fungal Airway Microbiome in Cystic Fibrosis and Non- Cystic Fibrosis Bronchiectasis

**Leah Cuthbertson*^1,2^, Imogen Felton*^1,2^, Phillip James*^1,2^, Michael J Cox^2^, Diana Bilton^1,2^, Silke Schelenz^1^, Michael R Loebinger^1,2^, William OC Cookson^1,2^¶†, Nicholas J. Simmonds^1,2^¶, Miriam F Moffatt^2^¶**

**Supplementary Methods**

**Patient Characteristics**

Patients attending the adult outpatient Host Defense clinic and CF clinic at the Royal Brompton Hospital were invited to take part with informed, written consent acquired from all subjects. Patients with bronchiectasis had all previously been screened according to the British Thoracic Society bronchiectasis guidelines.(1) Subjects in the bronchiectasis cohort were defined as having symptoms of chronic cough, sputum production and malaise related to high-resolution computed tomography (CT) evidence of airway wall thickening and dilatation and in which CF, primary ciliary dyskinesia and underlying immunodeficiency’s had been reasonably excluded.(2)

Subjects with CF had been diagnosed according to standard criteria whereby a sweat chloride level greater than or equal to 60 mmol/L and/or two CF-causing mutations had been identified.(3)

All subjects who were pregnant or participating in concurrent interventional trials were excluded.

Study subjects were partitioned into *a priori* defined clinical subgroups according to the study criteria listed in the on-line supplement (Table S1).

**Microbiological Culture**

Sputum samples from patients with bronchiectasis underwent standard clinical microbiology for non-CF sputum samples. Samples were plated onto chocolate blood agar, blood agar and MacConkey agar from Oxid. Chocolate blood agar and blood agar plates were incubated in 5% CO_2_ at 37 °C, while MacConkey plates were incubated in atmospheric conditions at 37 °C.

CF sputum was performed according to the local (Royal Brompton Hospital, Clinical Microbiology Laboratory [RBH-CML]) protocol. Sputum was liquified using equal volume of working Sputasol (Oxoid-SR0233A) solution mixed on the vortex then incubated at 35-37^o^C for 15 minutes. Sputum homogenate was diluted to 1:100 and 1:1000 in Ringers solution. 20μl aliquots from the 1:1000 dilution were inoculated on plates shown in Table S2 to produce discrete colonies, with the exception of Chocolate blood agar, which 20 μl of the 1:100 dilution was evenly inoculated and an Optochin disc was then placed centrally on the plate after which it was incubated in 5 % CO_2_ at 37 °C. As per RBH-CML protocol all the routine CF plates were incubated for up to 48 hrs with daily reading.

Fungal culture was carried out by inoculating Sabouraud agar with chloramphenicol and the Dermasal agar plates with 20 μl of the Sputum/Sputasol homogenate. Sabouraud agar and the Dermasal plates were inoculated and incubated for 2 days at 37 °C and then up to 28 days (4 weeks) at 30 °C, plates were read daily.

Slides were prepared to look for fungal elements and yeasts by pipetting 1-2 drops of 0.1 % Calcofluor white stain onto the slide. The slides were examined under a fluorescent microscope with a 420-490nm excitation filter and a 520nm barrier filter using the x40 objective for fungal elements and hyphae exhibiting bright blue-white fluorescence with typical morphology. Fungal cultures which had no growth at 7 days where ‘Fungal elements’ were seen on Calcofluor microscopy, Sabourad plates were re-incubated for a further 3 weeks i.e. 4 weeks incubation in total.

**Fungal 18S rRNA gene qPCR**

Briefly, primers 5-GGRAAACTCACCAGGTCCAG -3 and 5-GSWCTATCCCCAKCACGA 3 were used to amplify a 353 bp region of the pan fungal 18S rRNA gene. QPCR reactions were performed in triplicate on a Viia 7 real time PCR system (Applied Biosystems, Paisley, UK) in reactions consisting of 7.5 μl Low Rox Kapa Probe Fast qPCR kit (Kappa Bioscience, Oslo, Norway), 1.8 μM of each primer, 1 % formamide, 5 μl of 1 in 5 diluted template and made up to 15 μl with ultra-pure, microbiologically clean certified water (Cambio, Cambridge, UK). A FAM labelled probe with a minor grove binding non-florescent quencher ((6FAM) 5-TGGTGCATGGCCGTT-3 (MGBNFQ)) at 225 nM was used to detect amplification of fungal specific 18S rRNA gene sequences. Cycling conditions consisted of an initial denaturation step of 95 °C for 2 minutes followed by 50 cycles of 95 °C for 15 second, 65 °C for 1 minute.

QPCR standards were generated from a cloned partial 18S rRNA gene belonging to the *C. albicans* type strain (ATCC MYA-2876D-5) in PCR-4 vector (Life Technologies Ltd, Paisley, UK). Plasmids were quantified using Quantit picogreen dsDNA Assay kit (Promega, Madison, USA) and serially diluted 10 fold to form standards ranging from 1 x 10^6^ to 1 x 10^1^. Triplicate no template controls were also included with 5 μl of microbiologically clean ultrapure water (Cambio, Cambridge, UK). Resultant quantities were normalised to represent copy number per g of sputum.

**Fungal ITS2 Sequencing**

For each extracted DNA sample, the ITS2 region of the fungal genome was amplified by PCR using indexed and MiSeq adaptor ligated pan fungal primers 3271-ITS2-F (5’-CAR CAA YGG ATC TCT TGG-3’) and 3271-ITS2-R (5’-GAT ATG CTT AAG TTC AGC GGG T-3’) (Table S3).(4) Extraction controls and a mock community of known ITS2 genes (Table S4) were also amplified and sequenced concurrently. To generate the mock community, ITS2 genes from type strain genomic DNA (LGC, Middlesex, UK) were cloned into PCR 2.1 vectors using the Topo TA cloning kit and high competency *E. coli* (Life Technologies Ltd, Paisley, UK) according to the manufacturer’s instructions. Vectors containing ITS2 genes from each fungal organism were pooled in equi-molar concentrations to form the mock community. One mock community control sample was included on each MiSeq run. Batch effects were tested for using a permutational multivariate ANOVA (*P* > 0.01) and comparison of the mock community composition. Samples and extraction controls were randomly distributed across all runs. PCR reactions were comprised of 12.5 µl 2x Q5 master-mix (NEB, Ipswich, MA, USA), 0.5 µM of each primer, 1µl of template and made up to 25µl with certified microbiologically sterile water (Cambio, Cambridge, UK). PCR cycling conditions were 98 °C for 1 min, followed by 35 cycles of; 98 °C for 10 s; 55 °C for 30 s; 72 °C for 30 s; then a final extension of 72 °C for 2 min. PCR negative controls were visually inspected on a 1.2 % agarose gel with 1 x Gel Red (Bio-Rad Labs Ltd, Hemel Hempstead, UK) to ensure no PCR contamination was detectable. Primer dimers were removed using Ampure XP beads (Life Technologies Ltd, Paisley, UK) at a 0.7:1 ratio and purified amplicons were quantified using the picogreen dsDNA quantification assay (Life Technologies Ltd, Paisley, UK) in preparation for equi-molar pooling.

Pooled amplicons were concentrated using Ampure bead selection as before then purified using continuous gel selection of bands ranging in size from 300 – 700 bp in length. Purified libraries were examined for adaptor dimer using a High sensitivity DNA chip (Agilent Technologies, Waldbronn, Germany) on a Bioanalyzer 2100 prior to quantification by qPCR using an Illumina library quantification kit (Kapa Biosystems Ltd. London, UK). Indexed samples including a 20 % spike of Phix control DNA (Illumina, Cambridge, UK) was sequenced on the MiSeq (Illumina, Cambridge, UK) using a V3 kit, generating bidirectional 300 bp reads. Custom sequencing primers R1 (5’-CCC GGG CAC GTT TCA RCA AYG GAT CTC TTG G-3’), R2 (5’-ACC CGC TGA ACT TAA GCA TAT CTG ACT GAC CGA CT-3’) and R4 (5’-AGT CGG TCA GTC AGA TAT GCT TAA GTT CAG CGG GT-3’) (Table S5) were spiked into the cartridge prior to sequencing.

**Sequence Processing**

Barcodes were extracted from each sequence using QIIME 1.9.0 (5) and concatenated into 16 bp unique sample identifiers. Prior to joining bidirectional sequences, primer sequence trimming and low quality truncation (< Q20) was performed using Trim Galore (https://github.com/FelixKrueger/TrimGalore). Pairs of sequences where either one was quality truncated to < 150 bp in length were removed from subsequent analysis. Surviving pairs were joined using fastq-join where the minimum overlap had to exceed 150 bp with less than 10 % mismatch. Samples were de-multiplexed using QIIME 1.9.0 where the maximum number of bases falling consecutively below Q30 was 10 and >70 % of the entire read had to be consecutively above Q30. PhiX contamination was removed by aligning all sequences against the PhiX genome(6) using BWA (7) where unaligned reads were retained. OTU generation was performed using UCLUST (8) in an open reference style. A pre-filter of 60 % homology to the UNITE ITS2 database (9) was used prior to OTU generation against the UNITE database clustered at 97 % similarity.

Following this, sequences that failed to match the database were clustered into OTUs in a *de novo* fashion using UCLUST.(8) A representative sequence from each OTU was chosen based upon abundance within that OTU and taxonomy was assigned using the RDP naïve Bayesian classifier(10) against the QIIME compatible UNITE database. A single R object was created from the BIOM formatted OTU table, representative sequences and associated clinical metadata using phyloseq (version 1.30.0)(11) for subsequent analysis in R version. All statistical and ecological analyses were performed in R using the packages phyloseq(11), vegan (version 2.5-6)(12), dplyr (version 0.8.5)(13), ggplot2 (version 3.3.0)(14) and reshape2 (version 1.4.3)(15). OTUs with less than 20 sequences across all samples were removed and all samples were randomly sub-sampled to 1000 sequences for diversity and dissimilarity calculations.

**Controlling for DNA Extraction Kit Contamination**

This study was carefully controlled to minimize contamination through the inclusion of Mock communities, extraction controls and PCR negative controls on each sequencing run.

After initial sequencing processing through QIIME data was transferred to R for all further analysis. To identify potential contaminants within the dataset the Decontam package version 1.4.0 in R was using both the prevalence and frequency methods.(16) The prevalence method uses the presence/absence of OTUs across all samples and compares them to their presence/absence in negative controls, while the frequency method identifies OTUs found to increase in sequence read abundance with decreasing DNA concentration, determined by 18S rRNA gene qPCR. The batch functionality was used to explore the batch effects of extraction kits numbers.

After exploration of the data using all methods, no OTUs were identified as being associated with extraction kit using the “batch” functionality. All potential contaminant OTUs found to be associated with DNA concentration were found to have low prevalence and therefore the relationships were unconvincing. This may be due to the poor sensitivity of the 18S rRNA qPCR assay.

Using the prevalence method with a threshold of 0.1 identified 33 OTUs (Table S6) most likely sources of contamination in the data set. By using a threshold of 0.1, therefore the OTU must obtain a statistic probability of 0.1 to be considered a contaminant. This means sequences that are statistically more prevalent in negative controls are identified and removed from further analysis. These OTUs were removed from further analysis.

In this study several of the OTUs removed were identified as known fungal respiratory pathogens. Further investigation into these OTUs showed they were in very low prevalence and abundance. It was speculated that these OTUs were a result of sequencing errors and barcode switching rather than strain level variation. Inclusion of these OTUs in further analysis would not impact the overall results, however to provided a clear and open overview of the decontamination process these OTUs were removed from further analysis and shown in Table S6.

Singletons were removed from the dataset in line with published data which has shown that removal of singletons reduces the effect of chimeric sequences and decreases differences in diversity observed between repeat samples.(17)

*Batrachochytrium dendrobatidis* is a chytrid fungus of amphibians, this fungal species was used to examine the extent of cross contamination as it would not be expected in the sequencing runs. Only 6.25% (n = 11) of the samples had no *B. dendrobatidis* reads present. A maximum of 327,086 reads with a mean of 3885.7 and median of 38 *B.* *dendrobatidis* reads were found in the dataset. The total number of reads per samples was not related to number of *B.* *dendrobatidis* reads. Position on the plate was not related to number of contaminant reads. All *B. dendrobatidis* reads were removed from further analysis, this could not be used to remove other contamination but highlighted the requirement for careful assessment of OTUs in further analysis.

In line with QIIME recommendations samples with less than 1000 reads were removed from sequencing analysis as they are known to be unreliable for diversity analysis, samples were then rarefied to 1000 reads.

**Statistical Analysis**

Alpha diversity calculations were calculated using phyloseq version 1.30.0 (11), while Pielou’s evenness score was calculated as the Shannon index divided by the log of the OTU richness.

Sample dissimilarity and similarity statistics were calculated using Bray-Curtis distance and UPGMA hierarchical clustering where needed.

Indicator species analysis(18) was performed on rarefied data while differential abundance analysis was performed using DESeq2(19) (upon non-rarefied data (all samples > 30 sequences) but underwent sequence count normalization as part of the DESeq2 analysis.

**Differential abundance analysis & Indicator Taxa analysis**

DESeq2(19) and indicator species analysis(18) were used to identify OTUs significantly associated with disease groups. The former uses a matrix of non-rarefied count data per OTU (i.e. the OTU table) that has been pruned to remove any sequences with less than 1000 reads. This count data is normalized then, using a negative binomial generalized model, the log fold change of each OTU is calculated between two experimental groups. In this case, a group with underlying fungal/NTM disease and a control group classified as no active fungal disease.

Indicator taxa analysis calculates the association of a particular OTU with any experimental group based on presence absence data. Two values are computed, those being the positive prevalence and the fidelity.(18) An association statistic is calculated using a permutation test to define a statistical level of significance between OTUs and each experimental group in relation to all other experimental groups rather than a single control group.

**Supplementary Tables**

**Table S1**: Diagnosis criteria applied to disease groups.

| **Diagnosis** | **Diagnostic Criteria** |
| --- | --- |
| Allergic bronchopulmonary aspergillosis (ABPA) in non-CF bronchiectasis | Asthma |
|  | Skin reactions* |
|  | Raised total IgE in serum (>1000 ng/L)* |
|  | Raised specific IgE and/or IgG to *Aspergillus fumigatus* in serum* |
|  | Bronchiectasis* |
|  | Radiologically confirmed infiltrate |
|  | Eosinophilia in blood (>500AL) |
|  | Precipitating antibodies to Aspergillus fumigatus (modified from Agarwal *et al.* 2013)(20) |
|  | *Obligatory criterion for defining the diagnosis. |
| ABPA in CF | *Classic diagnostic criteria* |
|  | Acute or subacute clinical deterioration (cough, wheeze, and other pulmonary symptoms) not explained by another aetiology |
|  | Serum total IgE levels >1,000 IU/mL |
|  | Immediate cutaneous reactivity to Aspergillus or presence of serum IgE antibody to *A. fumigatus* Precipitating antibodies to *A. fumigatu*s or serum IgG antibody to *A. fumigatus* |
|  | New or recent abnormalities on chest radiograph or chest CT scan that have not cleared with antibiotics and standard physiotherapy |
|  | *Minimal diagnostic criteria* |
|  | Acute or subacute clinical deterioration (cough, wheeze, and other pulmonary symptoms) not explained by another aetiology |
|  | Total serum IgE levels >500 IU/mL. If total IgE level is 200-500 IU/mL, repeat testing in 13 mo is recommended Immediate cutaneous reactivity to Aspergillus or presence of serum IgE antibody to *A. fumigatus* |
|  | One of the following: |
|  | • Precipitins to *A. fumigatus* or demonstration of IgG antibody to *A. fumigates* |
|  | • New or recent abnormalities on chest radiography (on chest radiography or chest CT scan that have not cleared with antibiotics and standard physiotherapy)(Stevens et al 2003)(21) |
|  | Clinical: Chronic (>1 month) pulmonary or systemic symptoms, including at least one of: weight loss, productive cough or haemoptysis No overt immunocompromising conditions (e.g. haematological malignancy, neutropenia, organ transplantation) |
| Chronic necrotizing pulmonary aspergillosis (CNPA) | Radiological: Cavitary pulmonary lesion with evidence of paracavitary infiltrate |
|  | New cavity formation, or expansion of cavity size over time |
|  |  |
|  | Laboratory: Elevated levels of inflammatory markers (C-reactive protein, plasma viscosity or erythrocyte sedimentation rate). Isolation of Aspergillus spp. from pulmonary or pleural cavity, or positive serum Aspergillus precipitin test. Exclusion of other pulmonary pathogens, by results of appropriate cultures and serological tests, that are associated with similar disease presentation, including mycobacteria and endemic fungi. * |
|  | *Adapted from Denning *et al.* 2016.(22) |
| Fungal bronchitis (FB) | Acute or subacute deterioration characterized by increased cough, and/or productive of dark sputum/plugs, and/or positive sputum fungal markers* |
|  | Decreased lung function not attributable to another aetiology, and in the presence of elevated precipitating antibodies to A. *fumigatus IgG* (>90g/dL)**, and/or with favourable response to anti-fungal therapy trial. |
|  | *Sputum fungal markers = prolonged fungal cultures (<28 days), sputum microscopy with calcofluor white stain and cytology with Grocotts methenamine silver stain for characteristic fungal morphology, sputum galactomannan index assay. |
|  | ** Presence of elevated titers of total IgE (level >20 IU/mL) and Aspergillus-specific IgE (fluoroenzyme immunoassay [BAST]) (>0.35 Ua/mL) (Immunocap; Pharmacia) in serum but not reaching diagnostic criteria for ABPA. |
| No active fungal disease (NAFD) | Patients with either CF or non-CF bronchiectasis who did not have any clinical signs or symptoms of active fungal disease (e.g. FB or ABPA), in the presence of either positive or negative sputum fungal cultures. |
| Non-tuberculous mycobacteria (NTM) | Patients with either CF or non-CF bronchiectasis who met criteria for anti-NTM treatment (Haworth *et al.* 2017)(23), or whom were receiving anti-NTM therapy*. |
|  | *NTM infection was deemed the primary diagnosis above co-existing fungal lung disease if also present. |

**Table S2**: Culture conditions by media type.

| Agar Type | Media Catalogue Number | Temperature | Oxygen conditions | Time |
| --- | --- | --- | --- | --- |
| Pseudomonas | OXOIDPO0185A | 37 °C | Aerobic | 48hrs |
| MacConkey | OXOIDPO0148A | 37 °C | Aerobic | 48hrs |
| Mannitol salt | OXOIDPO0151A | 37 °C | Aerobic | 48hrs |
| *B.cepacia* media | OXOIDPO0938A | 37 °C for 5 days | Aerobic | 5 days |
| Chocolate blood | OXOIDPO0124A | 37 °C | 5% CO_2_ | 48hrs |
| Chocolate | OXOIDPB0220A | 37 °C | 5% CO_2_ | 48hrs |
| Sabouraud | OXOIDPO0737A | 37 °C for 48hrs. 30 °C for up to 28 days | Aerobic | Up to 28 days |
| Dermasal | OXOIDPO0737A | 37 °C for 48hrs. 30 °C for up to 28 days | Aerobic | Up to 28 days |

**Table S3**: Primer sequences with adaptor, index, pad and link sequences of the forward primer 3271-ITS2-F and the revere primer 3271-ITS2-R.

| **Index name** | **Direction** | **Adaptor** | **Index** | **Pad** | **Link** | **Primer** |
| --- | --- | --- | --- | --- | --- | --- |
| N501 | Forward | AATGATACGGCGACCACCGAGATCTACAC | TAGATCGC | CCCGGGCACG | TTT | CARCAAYGGATCTCTTGG |
| N502 | Forward | AATGATACGGCGACCACCGAGATCTACAC | CTCTCTAT | CCCGGGCACG | TTT | CARCAAYGGATCTCTTGG |
| N503 | Forward | AATGATACGGCGACCACCGAGATCTACAC | TATCCTCT | CCCGGGCACG | TTT | CARCAAYGGATCTCTTGG |
| N504 | Forward | AATGATACGGCGACCACCGAGATCTACAC | AGAGTAGA | CCCGGGCACG | TTT | CARCAAYGGATCTCTTGG |
| N505 | Forward | AATGATACGGCGACCACCGAGATCTACAC | GTAAGGAG | CCCGGGCACG | TTT | CARCAAYGGATCTCTTGG |
| N506 | Forward | AATGATACGGCGACCACCGAGATCTACAC | ACTGCATA | CCCGGGCACG | TTT | CARCAAYGGATCTCTTGG |
| N507 | Forward | AATGATACGGCGACCACCGAGATCTACAC | AAGGAGTA | CCCGGGCACG | TTT | CARCAAYGGATCTCTTGG |
| N508 | Forward | AATGATACGGCGACCACCGAGATCTACAC | CTAAGCCT | CCCGGGCACG | TTT | CARCAAYGGATCTCTTGG |
| N701 | Reverse | CAAGCAGAAGACGGCATACGAGAT | TAAGGCGA | AGTCGGTCAG | TCA | GATATGCTTAAGTTCAGCGGGT |
| N702 | Reverse | CAAGCAGAAGACGGCATACGAGAT | CGTACTAG | AGTCGGTCAG | TCA | GATATGCTTAAGTTCAGCGGGT |
| N703 | Reverse | CAAGCAGAAGACGGCATACGAGAT | AGGCAGAA | AGTCGGTCAG | TCA | GATATGCTTAAGTTCAGCGGGT |
| N704 | Reverse | CAAGCAGAAGACGGCATACGAGAT | TCCTGAGC | AGTCGGTCAG | TCA | GATATGCTTAAGTTCAGCGGGT |
| N705 | Reverse | CAAGCAGAAGACGGCATACGAGAT | GGACTCCT | AGTCGGTCAG | TCA | GATATGCTTAAGTTCAGCGGGT |
| N706 | Reverse | CAAGCAGAAGACGGCATACGAGAT | TAGGCATG | AGTCGGTCAG | TCA | GATATGCTTAAGTTCAGCGGGT |
| N707 | Reverse | CAAGCAGAAGACGGCATACGAGAT | CTCTCTAC | AGTCGGTCAG | TCA | GATATGCTTAAGTTCAGCGGGT |
| N708 | Reverse | CAAGCAGAAGACGGCATACGAGAT | CAGAGAGG | AGTCGGTCAG | TCA | GATATGCTTAAGTTCAGCGGGT |
| N709 | Reverse | CAAGCAGAAGACGGCATACGAGAT | GCTACGCT | AGTCGGTCAG | TCA | GATATGCTTAAGTTCAGCGGGT |
| N710 | Reverse | CAAGCAGAAGACGGCATACGAGAT | CGAGGCTG | AGTCGGTCAG | TCA | GATATGCTTAAGTTCAGCGGGT |
| N711 | Reverse | CAAGCAGAAGACGGCATACGAGAT | AAGAGGCA | AGTCGGTCAG | TCA | GATATGCTTAAGTTCAGCGGGT |
| N712 | Reverse | CAAGCAGAAGACGGCATACGAGAT | GTAGAGGA | AGTCGGTCAG | TCA | GATATGCTTAAGTTCAGCGGGT |

**Table S4**: Composition of the fungal mock community.

| **Fungal Species** | **ATCC Number** |
| --- | --- |
| *Candida albicans* | MYA-2876D-5 |
| *Candida albicans* | 66027D-5 |
| *Candida parapsilosis* | 22019D-5 |
| *Candida glabrata* | 2001D-5 |
| *Aspergillus flavus* | 9643D-2 |
| *Aspergillus versicolor* | 11730D-2 |
| *Aspergillus fumigatus* | 1022D-2 |
| *Aspergillus nidulans* | 38163D-2 |
| *Cryptococcus neoformans* | MYA-565D-5 |
| *Trichosporon mucoides* | 204094D-5 |
| *Malassezia restricta* | MYA-4611D-5 |
| *Fusarium solani* | 36031D-2 |
| *Penicillium marneffei* | 18224D-2 |

**Table S5**: Custom sequencing primers for ITS2 sequencing.

|  | **Primer** | |
| --- | --- | --- |
| **Read 1 Sequencing primer** | | CCCGGGCACGTTTCARCAAYGGATCTCTTGG |
| **Read 2 Sequencing Primer** | | ACCCGCTGAACTTAAGCATATCTGACTGACCGACT |
| **Read 4 Sequencing Primer** | | AGTCGGTCAGTCAGATATGCTTAAGTTCAGCGGGT |

**Table S6**: OTUs removed from the dataset using the prevalence method in Decontam package in R.

| **OTUID** | **Frequency** | **Prevalence** | ***P*** | **Contaminant** |
| --- | --- | --- | --- | --- |
| Hanseniaspora_thailandica_32 | 1.85E-04 | 16 | 0.086 | TRUE |
| Candida_xylopsoci_156 | 2.05E-03 | 19 | 0.008 | TRUE |
| Candida_blankii_611 | 1.12E-05 | 4 | 0.022 | TRUE |
| Candida_parapsilosis_767 | 5.20E-06 | 2 | 0.011 | TRUE |
| Talaromyces_marneffei_813 | 6.89E-03 | 207 | 0.071 | TRUE |
| Penicillium_psychrosexualis_1136 | 1.25E-05 | 12 | 0.064 | TRUE |
| Trichocomaceae_sp_1302 | 9.50E-06 | 12 | 0.064 | TRUE |
| Candida_glabrata_1324 | 3.06E-06 | 10 | 0.054 | TRUE |
| Aspergillus_subversicolor_1779 | 7.70E-06 | 9 | 0.048 | TRUE |
| Microascaceae_sp_1798 | 2.19E-05 | 12 | 0.064 | TRUE |
| Aspergillus_1855 | 5.76E-06 | 9 | 0.048 | TRUE |
| Candida_albicans_2088 | 9.22E-06 | 9 | 0.048 | TRUE |
| Candida_albicans_2484 | 8.52E-05 | 68 | 0.091 | TRUE |
| Leptosphaerulina_chartarum_2485 | 6.86E-04 | 18 | 0.097 | TRUE |
| Candida_glabrata_2528 | 7.06E-06 | 15 | 0.081 | TRUE |
| Trichocomaceae_3105 | 6.22E-06 | 8 | 0.043 | TRUE |
| Candida_albicans_3467 | 4.98E-06 | 6 | 0.032 | TRUE |
| Aspergillus_3708 | 1.01E-05 | 12 | 0.064 | TRUE |
| Lecanoromycetes_3749 | 4.60E-04 | 9 | 0.048 | TRUE |
| Candida_albicans_3800 | 2.63E-06 | 12 | 0.064 | TRUE |
| Candida_parapsilosis_4182 | 4.04E-06 | 2 | 0.011 | TRUE |
| Candida_glabrata_4354 | 1.23E-05 | 7 | 0.038 | TRUE |
| Candida_albicans_4506 | 2.62E-06 | 7 | 0.038 | TRUE |
| Scedosporium_apiosperma_4782 | 7.37E-06 | 9 | 0.048 | TRUE |
| Aspergillus_5737 | 1.49E-05 | 10 | 0.054 | TRUE |
| Candida_dubliniensis_5821 | 2.83E-06 | 3 | 0.016 | TRUE |
| Scedosporium_boydii_5946 | 5.45E-06 | 10 | 0.054 | TRUE |
| Trichocomaceae_sp_5948 | 1.06E-05 | 13 | 0.070 | TRUE |
| Candida_albicans_6077 | 2.85E-06 | 10 | 0.054 | TRUE |
| Candida_glabrata_9507 | 3.53E-06 | 10 | 0.054 | TRUE |
| Candida_orthopsilosis_10173 | 6.12E-06 | 3 | 0.016 | TRUE |
| Scedosporium_11037 | 6.14E-06 | 3 | 0.016 | TRUE |
| Candida_albicans_11669 | 3.20E-06 | 12 | 0.064 | TRUE |

**Table S7**: Samples removed due to low sequencing depth. Due to time constraints on the project it was not possible to repeat the sequencing for these samples.

|  | **Bronchiectasis** | **CF** | ***P*** |
| --- | --- | --- | --- |
| *n* | 10 | 20 |  |
| Group (%) |  |  | 0.298 |
| ABPA | 4 (40.0) | 5 (25.0) |  |
| CNPA | 1 (10.0) | 0 ( 0.0) |  |
| FB | 0 ( 0.0) | 2 (10.0) |  |
| NAFD | 3 (30.0) | 11 (55.0) |  |
| NTM | 2 (20.0) | 2 (10.0) |  |
| Fungal culture (%) | 0 ( 0.0) | 5 (25.0) | 0.264 |
| *Candida spp.* (%) | 0 ( 0.0) | 2 (10.0) | 0.848 |
| *Aspergillus fumigatus* (%) | 0 ( 0.0) | 2 (10.0) | 0.848 |
| *Exophiala dermatitidis* (%) | 0 ( 0.0) | 1 ( 5.0) | 1 |
| Number of reads (mean (SD)) | 471.70 (346.22) | 443.10 (199.98) | 0.775 |
| Sample ID |  |  | 0.414 |
| FAME00002 | 0 | 1 |  |
| FAME00003 | 1 | 0 |  |
| FAME00005 | 0 | 1 |  |
| FAME00013 | 0 | 1 |  |
| FAME00015 | 0 | 1 |  |
| FAME00016 | 0 | 1 |  |
| FAME00017 | 0 | 1 |  |
| FAME00019 | 0 | 1 |  |
| FAME00021 | 1 | 0 |  |
| FAME00026 | 1 | 0 |  |
| FAME00027 | 1 | 0 |  |
| FAME00028 | 1 | 0 |  |
| FAME00035 | 0 | 1 |  |
| FAME00041 | 0 | 1 |  |
| FAME00044 | 1 | 0 |  |
| FAME00047 | 1 | 0 |  |
| FAME00048 | 1 | 0 |  |
| FAME00049 | 0 | 1 |  |
| FAME00058 | 0 | 1 |  |
| FAME00064 | 1 | 0 |  |
| FAME00068 | 1 | 0 |  |
| FAME00079 | 0 | 1 |  |
| FAME00082 | 0 | 1 |  |
| FAME00090 | 0 | 1 |  |
| FAME00110 | 0 | 1 |  |
| FAME00116 | 0 | 1 |  |
| FAME00213 | 0 | 1 |  |
| FAME00377 | 0 | 1 |  |
| FAME00385 | 0 | 1 |  |
| FAME00454 | 0 | 1 |  |

**Supplementary Figures**

**
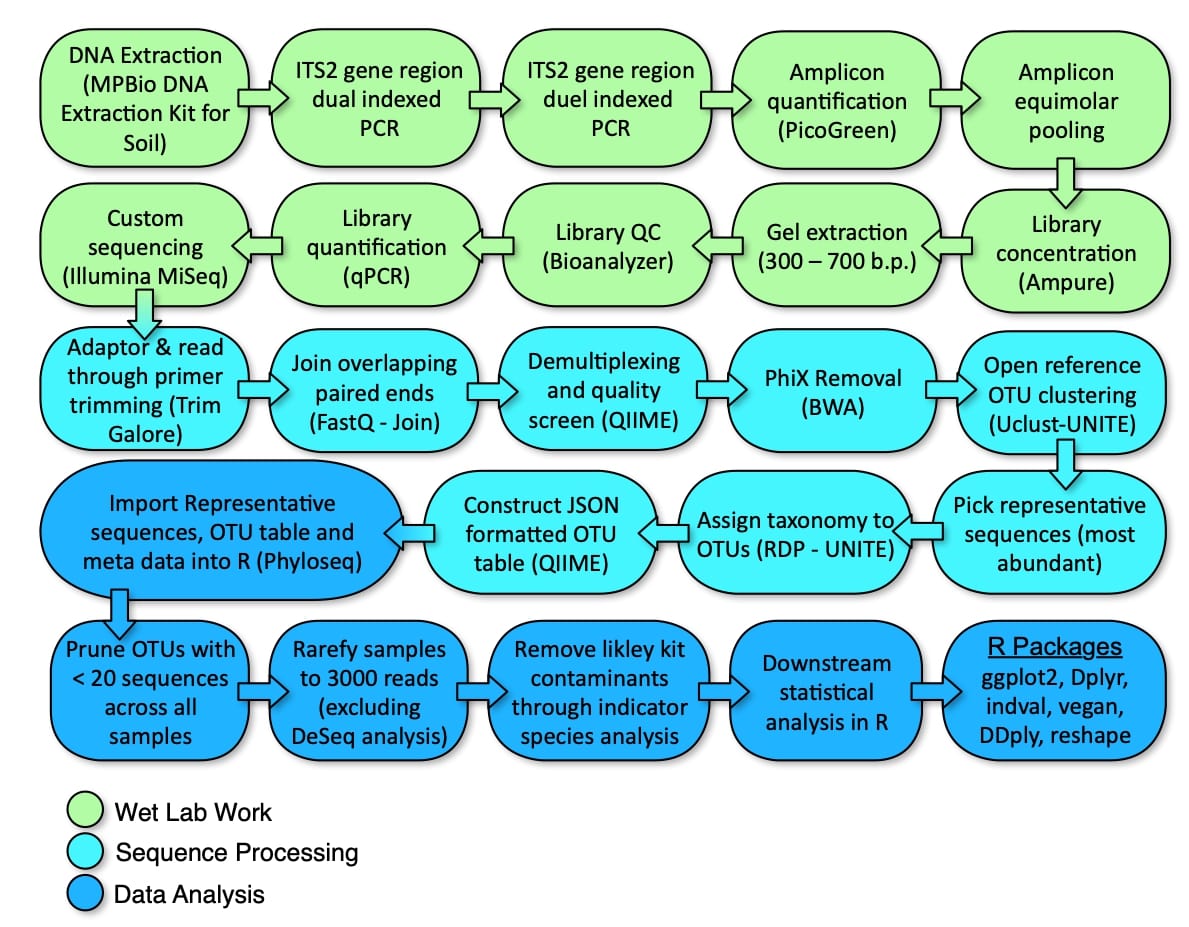
**

**Figure S1**. Schematic of sample processing, library preparation, and sequence pre-processing.

**Figure S2:** **Sequence dominance in detection of fungi by culture and/or sequencing (n=146).** The plot is split by number of samples positive for *Scedosporium* complex, *Exophiala dermatitidis*, *Aspergillus* spp or *Candida* spp. as determined by culture. Colours indicate if sample if positive for culture only or the most abundant OTU of each genus made up greater or less than 20% of the total reads in the sample.


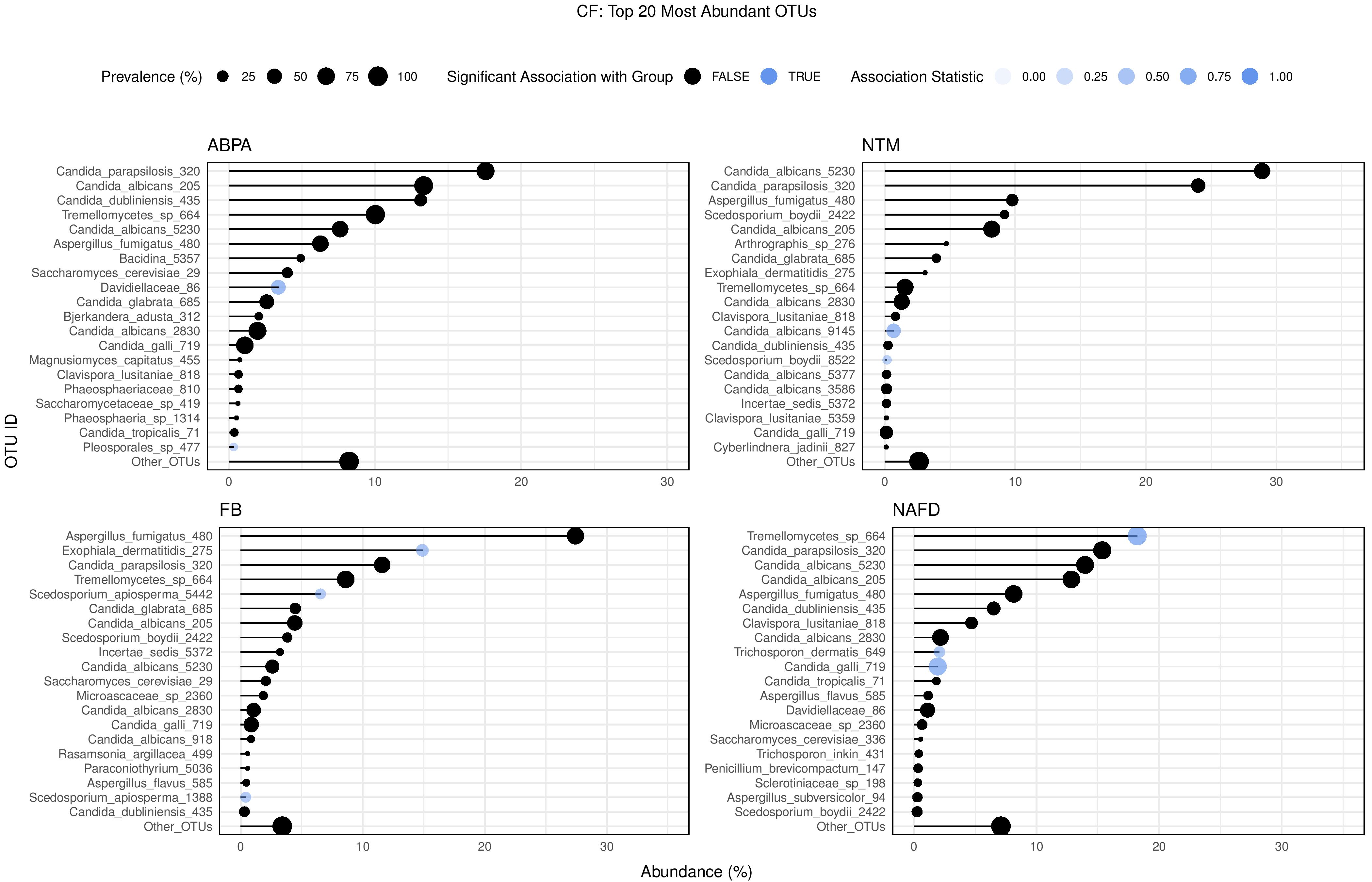


**Figure S3**: Abundance and prevalence of most abundant OTUs within the CF cohort split by disease subgroup (Table S1). Coloured points identify OTUs highlighted as significant by indicator taxa analysis while alpha value denotes the association statistic.


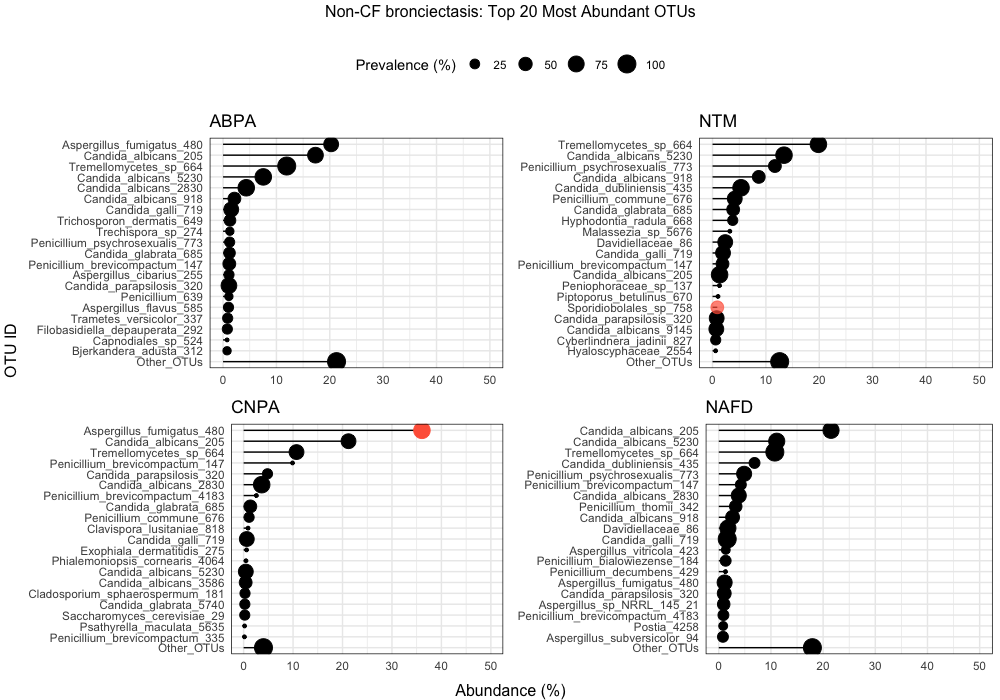


**Figure S4**: Abundance and prevalence of most abundant OTUs within the Bronchiectasis cohort split by disease subgroup. Red coloured points identify OTUs highlighted as significant by indicator taxa analysis while alpha value denotes the association statistic.


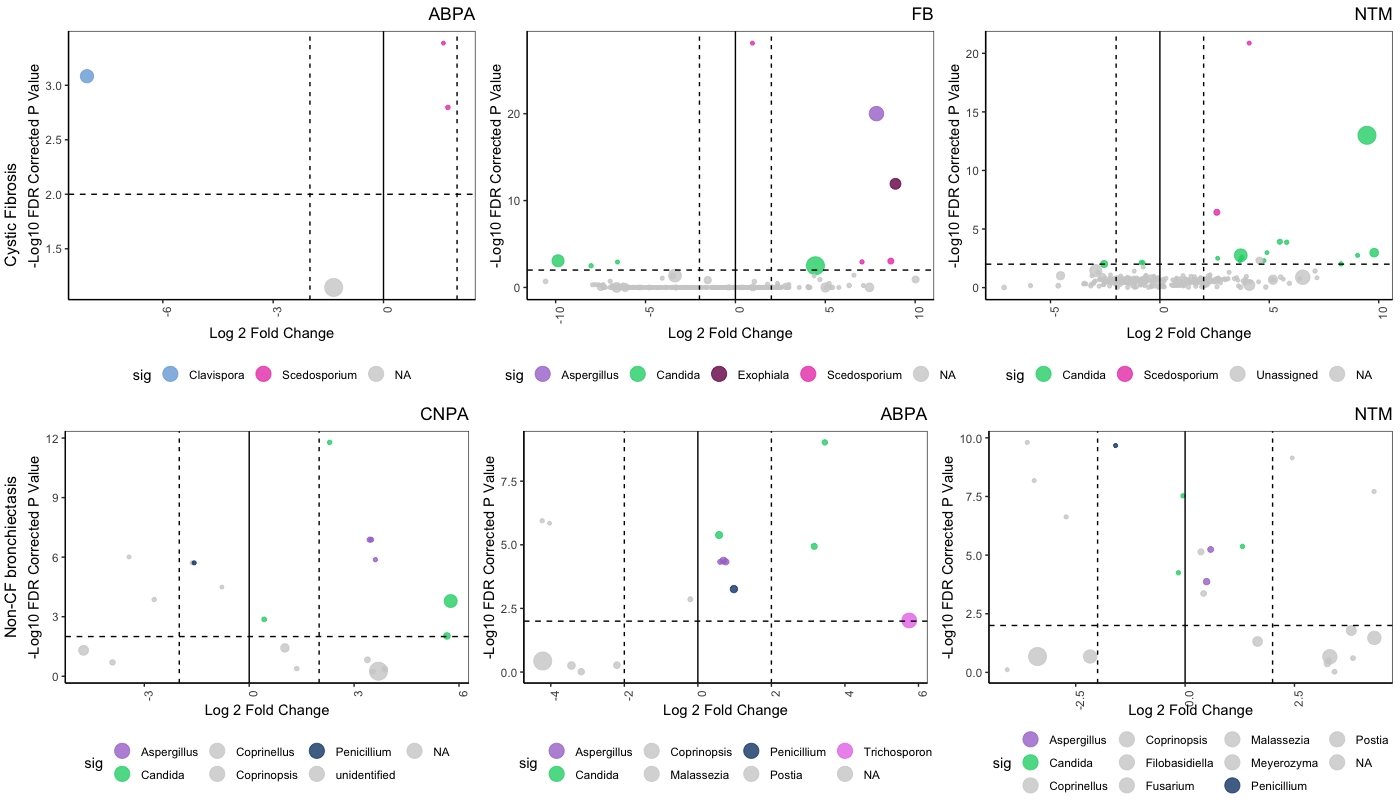


**Figure S5**: Differential abundance analysis by DESeq2 using shrinkage showing OTUs that have a significantly increased proportional abundance in fungal disease subgroups compared to the no fungal disease controls in the CF and bronchiectasis cohorts (BH corrected *P* < 0.01). Point size gives an indication of the number of sequences in each that OTU. Dashed vertical lines are included at -2 and 2 log 2 fold change for reference.

**Figure S6**: Boxplot of diversity measures (Simpsons, Shannon’s, Richness and Pielou’s evenness) between disease groups. All disease groups in non-CF bronchiectasis patients were significantly more diverse than those with CF. Within the CF group, fungal bronchitis (FB) was found to have the lowest diversity. FB was found to have significantly lower diversity than CF ABPA and those with no active fungal disease.


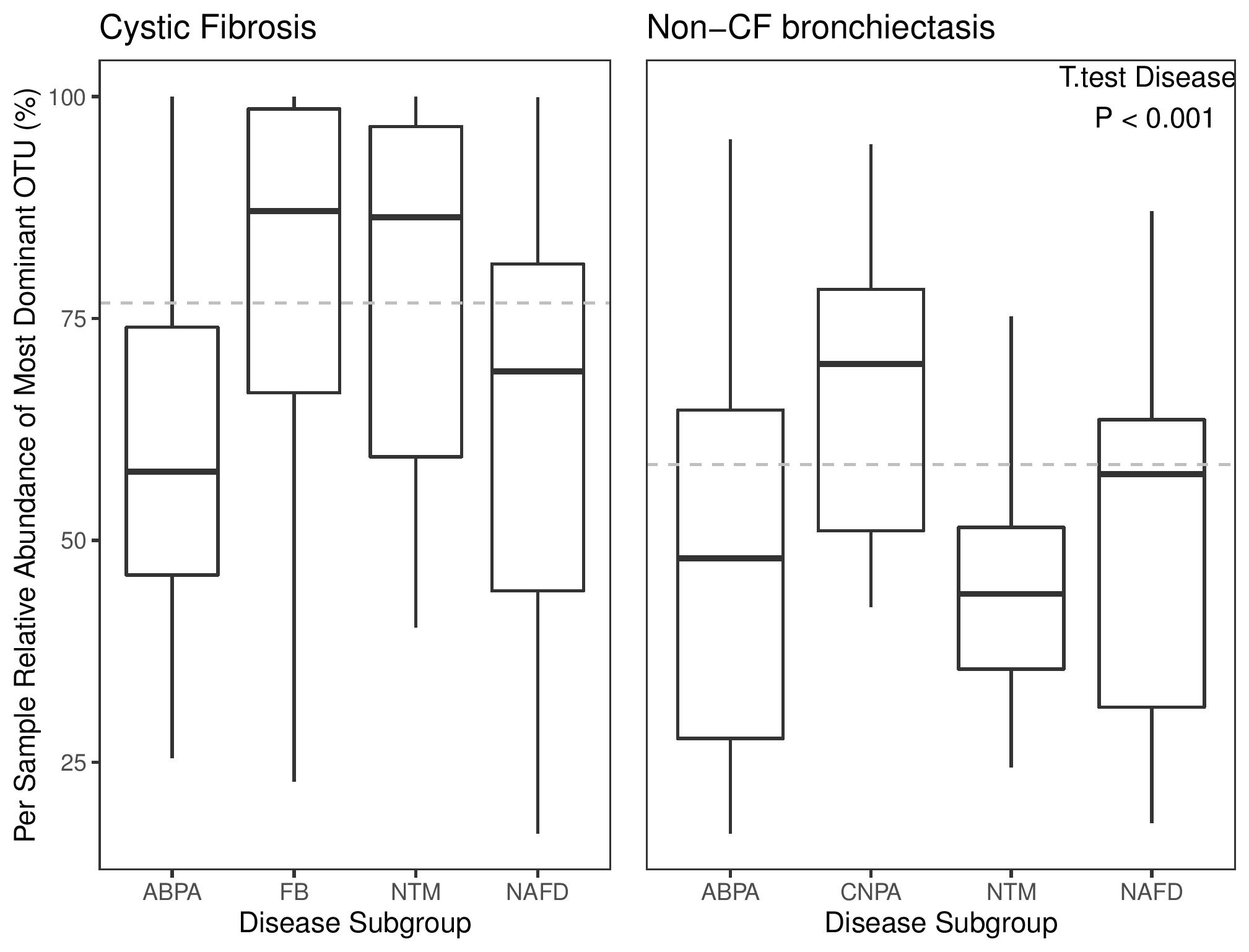


**Figure S7: Relative abundance of the most dominant OTU by disease subgroup. Samples from patients with CF were found to be more dominated than those with bronchiectasis who had a more diverse fungal community.** Semi-invasive fungal disease groups (CNPA and FB) were often dominated by single OTUs contributing upwards of 70 % of the sequences in each sample (see Figure 3).


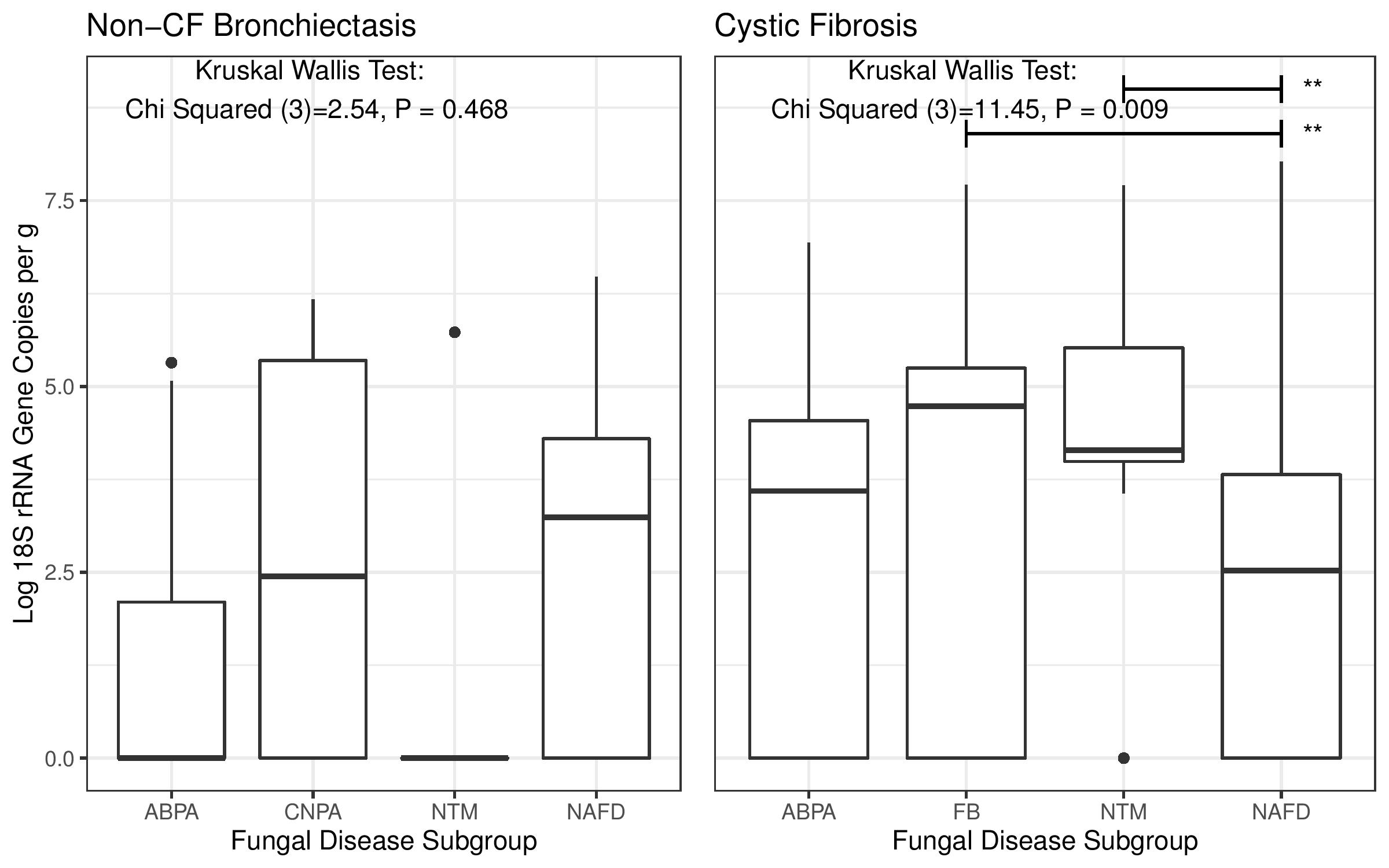


**Figure S8: Fungal 18S rRNA QPCR copies per g of sputum. There were significantly greater fungal 18S rRNA gene copies per gram of sputum in FB and NTM CF groups compared with the NAFD controls.**

**References**

1. Pasteur MC, Bilton D, Hill AT. British Thoracic Society guideline for non-CF bronchiectasis. Thorax. 2010;65 Suppl 1:i1-58.

2. Bilton D. Update on non-cystic fibrosis bronchiectasis. Current opinion in pulmonary medicine. 2008;14(6):595-9.

3. De Boeck K, Wilschanski M, Castellani C, Taylor C, Cuppens H, Dodge J, et al. Cystic fibrosis: terminology and diagnostic algorithms. Thorax. 2006;61(7):627-35.

4. Liu CM, Kachur S, Dwan MG, Abraham AG, Aziz M, Hsueh PR, et al. FungiQuant: A broad-coverage fungal quantitative real-time PCR assay. Bmc Microbiology. 2012;12.

5. Caporaso JG, Kuczynski J, Stombaugh J, Bittinger K, Bushman FD, Costello EK, et al. QIIME allows analysis of high-throughput community sequencing data. Nat Methods. 2010;7(5):335-6.

6. Air GM, Els MC, Brown LE, Laver WG, Webster RG. Location of antigenic sites on the three-dimensional structure of the influenza N2 virus neuraminidase. Virology. 1985;145(2):237-48.

7. Li H, Durbin R. Fast and accurate short read alignment with Burrows-Wheeler transform. Bioinformatics. 2009;25(14):1754-60.

8. Edgar RC. Search and clustering orders of magnitude faster than BLAST. Bioinformatics. 2010;26(19):2460-1.

9. Abarenkov K, Henrik Nilsson R, Larsson KH, Alexander IJ, Eberhardt U, Erland S, et al. The UNITE database for molecular identification of fungi--recent updates and future perspectives. New Phytol. 2010;186(2):281-5.

10. Wang Q, Garrity GM, Tiedje JM, Cole JR. Naive Bayesian classifier for rapid assignment of rRNA sequences into the new bacterial taxonomy. Appl Environ Microbiol. 2007;73(16):5261-7.

11. McMurdie PJ, Holmes S. phyloseq: an R package for reproducible interactive analysis and graphics of microbiome census data. PLoS One. 2013;8(4):e61217.

12. Jari Oksanen FGB, Michael Friendly, Roeland Kindt, Pierre Legendre, Dan McGlinn, Peter R. Minchin, R. B. O'Hara, Gavin L. Simpson, Peter Solymos, M. Henry H. Stevens, Eduard Szoecs, Helene Wagner. vegan: Community Ecology Package. R package version 2.5-4. <https://CRANR-projectorg/package=vegan>. 2019.

13. Hadley Wickham RF, Lionel Henry, Kirill Müller dplyr: A Grammar of Data Manipulation. R package version 0801. 2019;<https://CRAN.R-project.org/package=dplyr>.

14. Wickham H. ggplot2: Elegant Graphics for Data Analysis. Springer-Verlag New York. 2016.

15. Wickham H. Reshaping data with the reshape package. Journal of Statistical Software. 2007;21(12).

16. Davis NM, Proctor DM, Holmes SP, Relman DA, Callahan BJ. Simple statistical identification and removal of contaminant sequences in marker-gene and metagenomics data. Microbiome. 2018;6(1):226.

17. Auer L, Mariadassou M, O'Donohue M, Klopp C, Hernandez-Raquet G. Analysis of large 16S rRNA Illumina data sets: Impact of singleton read filtering on microbial community description. Mol Ecol Resour. 2017;17(6):e122-e32.

18. Dufrene M, Legendre P. Species assemblages and indicator species: The need for a flexible asymmetrical approach. Ecological Monographs. 1997;67(3):345-66.

19. Love MI, Huber W, Anders S. Moderated estimation of fold change and dispersion for RNA-seq data with DESeq2. Genome Biol. 2014;15(12):550.

20. Agarwal R, Chakrabarti A, Shah A, Gupta D, Meis JF, Guleria R, et al. Allergic bronchopulmonary aspergillosis: review of literature and proposal of new diagnostic and classification criteria. Clin Exp Allergy. 2013;43(8):850-73.

21. Stevens D, Moss R, Kurup V, Knutsen A, Greenberger P, Judson M, et al. Allergic bronchopulmonary aspergillosis in cystic fibrosis - State of the art: Cystic Fibrosis Foundation Consensus Conference. Clinical Infectious Diseases. 2003;37:S225-S64.

22. Denning D, Cadranel J, Beigelman-Aubry C, Ader F, Chakrabarti A, Blot S, et al. Chronic pulmonary aspergillosis: rationale and clinical guidelines for diagnosis and management. European Respiratory Journal. 2016;47(1):45-68.

23. Haworth C, Banks J, Capstick T, Fisher A, Gorsuch T, Laurenson I, et al. British Thoracic Society guidelines for the management of non-tuberculous mycobacterial pulmonary disease (NTM-PD). Thorax. 2017;72.
